# Behavioural and molecular characterisation of the Dlg2 haploinsufficiency rat model of genetic risk for psychiatric disorder

**DOI:** 10.1111/gbb.12797

**Published:** 2022-01-25

**Authors:** Sophie Waldron, Rachel Pass, Simonas Griesius, Jack R. Mellor, Emma S. J. Robinson, Kerrie L. Thomas, Lawrence S. Wilkinson, Trevor Humby, Jeremy Hall, Dominic M. Dwyer

**Affiliations:** ^1^ School of Psychology Cardiff University Cardiff UK; ^2^ Neuroscience and Mental Health Research Institute Cardiff University Cardiff UK; ^3^ Neurobiology Research Unit Okinawa Institute of Science and Technology Onna‐son Okinawa Japan; ^4^ Centre for Synaptic Plasticity, School of Physiology, Pharmacology and Neuroscience University of Bristol, University Walk Bristol UK; ^5^ Medical Research Council Centre for Neuropsychiatric Genetics and Genomics Cardiff University Cardiff UK

**Keywords:** animal models, autism spectrum disorder, DLG2, PCP locomotion, schizophrenia, sensorimotor gating, synaptic plasticity

## Abstract

Genetic studies implicate disruption to the *DLG2* gene in copy number variants as increasing risk for schizophrenia, autism spectrum disorders and intellectual disability. To investigate psychiatric endophenotypes associated with *DLG2* haploinsufficiency (and concomitant PSD‐93 protein reduction) a novel clinically relevant *Dlg2* ^*+/−*^ rat was assessed for abnormalities in anxiety, sensorimotor gating, hedonic reactions, social behaviour, and locomotor response to the N‐Methyl‐D‐aspartic acid receptor antagonist phencyclidine. *Dlg* gene and protein expression were also investigated to assess model validity. Reductions in PSD‐93 messenger RNA and protein were observed in the absence of compensation by other related genes or proteins. Behaviourally *Dlg2* ^*+/−*^ rats show a potentiated locomotor response to phencyclidine, as is typical of psychotic disorder models, in the absence of deficits in the other behavioural phenotypes assessed here. This shows that the behavioural effects of *Dlg2* haploinsufficiency may specifically relate to psychosis vulnerability but are subtle, and partially dissimilar to behavioural deficits previously reported in *Dlg2* ^*+/−*^ mouse models demonstrating issues surrounding the comparison of models with different aetiology and species. Intact performance on many of the behavioural domains assessed here, such as anxiety and reward processing, will remove these as confounds when continuing investigation into this model using more complex cognitive tasks.

## Graphical abstract

To investigate psychiatric endophenotypes associated with DLG2 haploinsufficiency (and concomitant PSD‐93 protein reduction) a novel clinically relevant *Dlg2* ^*+/−*^ rat was assessed for abnormalities in anxiety, sensorimotor gating, hedonic reactions, social behaviour, and locomotor response to the N‐Methyl‐D‐aspartic acid receptor antagonist phencyclidine. Reductions in PSD‐93 messenger RNA and protein were observed in the absence of compensation by other related genes or proteins. Behaviourally *Dlg2* ^*+/−*^ rats show a potentiated locomotor response to phencyclidine, as is typical of psychotic disorder models, in the absence of deficits in the other behavioural phenotypes assessed here.

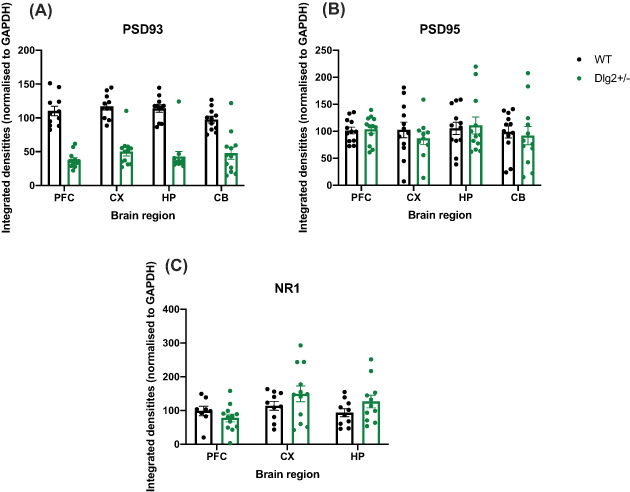

## 1 INTRODUCTION

The *DLG2* gene locus is linked to multiple psychiatric disorders. Point mutations in promotor regions have been associated with autism,^1^ schizophrenia and intellectual disability.^2^ Copy number variants encompassing complete deletion of one copy of *DLG2* result in increased risk for schizophrenia,^3^ autism,^4^ bipolar disorder^5^ and epilepsy.^6^ Such clinical evidence highlights the potential importance of *DLG2* in the psychopathologies common to a broad range of disorders. *DLG2* encodes the postsynaptic scaffold protein PSD‐93 (also known as Chapsyn‐110) in the membrane associated guanylate kinase (MAGUK) family. These proteins are responsible for anchoring and organising the numerous protein complexes required for development and plasticity at the synapse, in particular the NMDA receptor,^7^, ^8^, ^9^, ^10^ AMPA receptor,^11^ potassium ion channels^8^, ^12^, ^13^ and neuroligin 1–3.^7^

Previous investigation into specific behavioural endophenotypes driven by *Dlg2* disruption has used mouse models comprising both homozygous and heterozygous genetic lesions. Assessing homozygous knockdown models investigates the function of PSD‐93 and potential compensation mechanisms in its absence. In contrast, heterozygous models are faithful to the deletions seen in human psychiatric patients and allow investigation into endophenotypes which could increase disease risk under these circumstances. *Dlg2* ^*−/−*^ mice have shown abnormal social behaviours including impaired social preference,^14^ increased repetitive behaviours and hypoactivity in response to novelty.^14^, ^15^ Deficits in cognitive flexibility and attention have also been shown,^16^ aligning with similar deficits in human carriers of mutations to the *DLG2* coding region.

On some behavioural tasks the phenotypes of homozygous and heterozygous models align. Yoo et al.^14^ found that self‐grooming appeared to increase with *Dlg2* dosage, with the heterozygotes showing increased grooming relative to wild‐types and the homozygotes showing more grooming than the heterozygotes. Winkler et al.^15^ found a similar effect with reductions to *Dlg2* causing more severe impairments to motor learning and coordination. However, in these investigations altered social behaviour^14^, ^15^ and hypoactivity in response to novelty^14^, ^15^ seen in homozygous mutants were not found in heterozygotes. From a clinical perspective this could indicate that *Dlg2* heterozygosity does not increase psychiatric risk via altering social capabilities or habituation to stimuli, despite homozygous models indicating a role of PSD‐93 in normal performance of these behaviours. Phenotypic differences between homozygous and heterozygous mouse models led us to focusing on assessment of a heterozygous rat model with the aim of isolating processes that precipitate disease arising from *Dlg2* haploinsufficiency.

A range of psychiatric endophenotypes remain to be tested in heterozygous Dlg2 models, including faulty sensorimotor gating, anhedonia, and locomotor response to pharmacological challenge. This work presents the first molecular and behavioural characterisation of a rat model generated using CRISPR/Cas9 gene editing technology which contains only one copy of the *Dlg2* gene.

Here we assess whether the expected biological changes (reduced *Dlg2* mRNA and protein expression) occurred in the heterozygous (+/−) model, and whether there is evidence of compensation for possible *Dlg2* reduction from other MAGUK family members or related proteins, as has been shown in cortical neurons with complete PSD93 knockdown.^17^ Behavioural consequences of *Dlg2* haploinsufficiency was assessed by comparing *Dlg2* ^*+/−*^ rats and wild‐type littermates on a battery of psychiatric‐relevant translational tests. These included tests of anxiety, social behaviour, and anhedonia, key behavioural domains disrupted across disorders *Dlg2* is implicated in. Regulating unwanted or unnecessary sensory inputs was assessed using sensorimotor gating, deficiencies of which characterise patients with schizophrenia^18^, ^19^ and autism,^20^ in addition to rodent models of these conditions.^21^ Hyperlocomotion in response to PCP (an NMDAR‐antagonist) was also assessed as acute administration of PCP produces a transient psychosis‐like phenotype^22^, ^23^ which may be exaggerated in a rodent model with potential NMDAR alteration of function^24^ and an underlying genetic propensity towards psychosis.

*Dlg2* ^*+/−*^ rats demonstrated selective reductions in mRNA and protein expression that was not compensated for by increases in the remainder of the *Dlg* family. There was also an absence of gross behavioural deficits related to anxiety, hedonic reactions, social behaviour, and sensorimotor gating in the model; however, *Dlg2* ^*+/−*^ rats demonstrated a potentiated hyperlocomotion phenotype in response to PCP administration compared to wild‐types. Together, these confirm the biological validity of the current model selectively relevant to reduced expression of *Dlg2* and a possible concomitant alteration of NMDAR function^24^ and furthermore suggests that clinically relevant reductions to *Dlg2* expression will have more subtle effects than homozygous knockout mouse models.

## 2 MATERIALS AND METHODS

### 2.1 Animals

*Dlg2* heterozygous rats were generated on a Long Evans Hooded background by Horizon Discovery (Pennsylvania, USA) using CRISPR/Cas9 gene editing technology. Successful founders generated by Horizon Discovery had a 7 bp deletion (782933–782939 in the genomic sequence) in exon 5 which caused a frame shift and generation of an early stop codon in exon 6. Confirmation of successful non‐homologous end joining activity was assessed by PCR and sequenced by Horizon Discovery, UK. Selected heterozygous founders were send to Charles River (Margate, UK) and bred to produce experimental colonies by breeding male heterozygous rats were bred with female wild‐types resulting in a Mendelian distribution of wild‐type and heterozygous pups. A more detailed description of the generation of this rat line can be found in Supplement 1 of Griesius et al.^24^

Animals were housed in groups from two to four in standard cages (l × w × h: 50 cm × 32 cm × 21 cm) in rooms with a temperature between 19 and 23°C maintained on a 12 h light–dark cycle. Cages had sawdust and paper nesting and environmental enrichment (wooden chews and cardboard tubes). Food and water were given ad‐lib while conducting all tasks except the lick microstructure assessment where rats were maintained at 85–95% of their free‐feeding weight by giving them restricted access to food at the end of each day. Research was conducted in accordance with the Home Office regulations under the Animal (Scientific Procedures) Act 1986 Amendment Regulations (SI 2012/3039) under the authority of PPL 303243 or PPL 303135. Five cohorts of animals were used across the studies here, with all rodents participating in experiments aged between 2 and 6 months of age (see Table 1 for details of numbers in each genotype/sex group). Cohort 1—for assays elevated plus maze, open field and sensorimotor gating in that order; Cohort 2—for lick microstructure assessment; Cohort 3—for assays social preference and PCP hyperlocomotion in that order; Cohort 4—24 male for Western blot and Cohort 5—16 male for qPCR.

**TABLE 1:** Summary table of the sex and genotype of rodents used in the Experimental cohorts used. Not applicable (n/a) is used where no rats of this sex and genotype were used

Cohort	wild‐type male	*Dlg2* ^*+/*−^ male	wild‐type female	*Dlg2* ^*+/*−^ female
1	8	12	16	9
2	28	20	n/a	n/a
3	14	19	12	13
4	12	12	n/a	n/a
5	8	8	n/a	n/a

### 2.2 Tissue extraction

Rodents used for qPCR and Western blot were culled at 2–4 months old by inhalation of slowly rising CO_2_ concentration for 8 min (administered by Home Cage Culling Chamber, Clinipath Equipment Ltd, UK) 2 weeks after completion of the same set of behavioural experiments focusing on reward learning which are not reported here. Brains were extracted from the skull and gross dissected by partitioning the cerebellum and rostral‐most part of cortex (prefrontal cortex). The hippocampus and posterior cortex were then dissected. Extracted brain regions were flash frozen on dry ice and stored at ‐80°C prior to use.

### 2.3 Quantitative polymerase chain reaction (qPCR)

Using the QIAGEN RNeasy kit, RNA was isolated from prefrontal cortex, hippocampal and cerebellar tissue from individual animals (Cohort 5). Samples were DNAase treated using TURBO DNA‐free™ Kit (Ambion Life Technologies), following the recommended protocol. cDNA synthesis was performed using the RNA to cDNA EcoDry™ Premix (Random Hexamers) synthesis tubes (Clontech), heated at 42°C for 75 min, followed by 80°C for 15 min. qPCR was conducted with SensiMix SYBR Green (Bioline) on the StepOne Plus (Life Technologies; 1 cycle 95°C, 10 mins; 45 cycles of 95°C, 15 s and 60°C, 1 min; with melt curves conducted 55°C, 1 min; 95°C for 15 s). All qPCR samples were run in triplicate and the outcome was calculated using 2^‐ΔΔCt^ method, normalised to UBC and SDHA housekeeping genes. Primer sequences are given in Table 2.

**TABLE 2:** Primer sequences used in qPCR

Gene	Forward 5′‐	Reverse 5′‐
Dlg2	GGACATCCCCGGATTAGGTG	TGTAGTTTATTTCCTGCCTCGTGA
Dlg1	CCCAGATGGTGAGAGTGACG	AGTTACGTGCTTCAGGCCTTT
Dlg3	GTCTAATCGGGACTTCCCTGG	TGGAACTGCTTTCGCTGTCA
Dlg4	ACAACCAAGAAATACCGCTACCA	CCCCTCTGTTCCATTCACCTG
UBC	CTTTGTGAAGACCCTGAC	CCTTCTGGATGTTGTAGTC
SDHA	GCTCTTTCCTACCCGCTCAC	GTGTCATAGAAATGCCATCTCCAG

### 2.4 Western blot

Western blot analysis was conducted on hippocampal, prefrontal cortex, posterior cortex and cerebellar tissue from WT (*n* = 12) and HET (*n* = 12) animals. Each tissue sample was lysed in Syn‐PER lysis and extraction buffer (Thermo Fisher, UK) with mini protease inhibitor cocktail (Roche Diagnostics) and phosphatase inhibitor (Cell signalling, UK) according to description from manufacturer. After using BCA Assay kit to measure the total amount of protein in each sample, electrophoresis and blotting were carried out.

Gels (4–12% NuPAGE Bis‐Tris Midi, 45 well) were loaded with 40 μg of protein per well. Samples were added to Laemmli buffer at a 1:1 ratio and this mixture heated at 96°C for 5 min to denature protein–protein interactions and facilitate antibody bindings. Samples were arranged so that brain regions and genotypes were counterbalanced across gels with a WT standard used on each gel for comparison. Gels were run at room temperature in NuPAGE™ Running Buffer (Invitrogen, UK) at 85 V for 20 mins and then for a further hour at 115 V. Protein was then transferred to 0.45 μm pore size nitrocellulose membrane (Invitrogen, UK) at 85 V for 2 h 15 min at an ambient temperature of 4°C in NuPAGE™ Transfer Buffer (Invitrogen, UK) containing 10% 2‐propanol (ThermoFisher Scientific, UK). Membranes containing transferred protein were washed in Tris‐Buffered Saline (20 mM Tris, 150 mM NaCl, pH 7.6) with 0.1% Tween 20 (TBST) before blocking in 5% milk for 1 h at room temperature with gentle rocking.

Primary antibodies were diluted to appropriate concentrations in 5% milk and incubated with the membrane overnight at 4°C. These included rabbit anti‐PSD93 (1:1000, Cell Signalling Technology, USA), mouse anti‐NR1 (1:1000, Merck Millipore, UK), rabbit anti‐PSD95 (1:2000, Abcam, UK) and mouse anti‐GAPDH (1:5000, Abcam, UK). Membranes were then subject to 3 × 10 min TBST washes before incubation with the appropriate fluorescent IRDye 680RD secondary antibodies at 1:15,000 dilution in 5% milk at room temperature. After another series of TBST washes membranes were imaged on Odyssey CLx Imaging System (Li‐COR, Germany). Densiometric analysis of bands was performed using ImageLab 6.0 (https://imagej.nih.gov/ij/). The densities (with background subtracted) of the protein of interest were divided by the loading control densities for each sample to provide normalised values. Densities were then averaged by group. The hippocampal protein expression outlined here is also reported in Griesius et al.^24^ Supplementary Information.

### 2.5 Elevated plus maze

The elevated plus maze (EPM) consisted of two open arms (45 cm long × 10 cm wide), two closed arms (45 cm long × 10 cm wide × 30 cm high) and a middle (10 × 10 cm) compartment forming the shape of a plus sign, elevated 50 cm above the ground. The room was dimly lit, with the light level in the open arms 26 lux, and the light level in the closed arms 15.3 lux. Rats were habituated to the testing room for at least 1 h before individual testing. Each rat was placed in the middle compartment with its head facing an open arm and allowed to freely explore the apparatus for 5 min. Between tests the arena was cleaned with 70% ethanol. Each test was recorded by a camera mounted 120 cm above the maze and MP4 videos subsequently analysed for movement across the maze by Ethovision software (Version XT V 13, Noldus Information Technologies, Netherlands) (frame rat of 7/s). For the EPM, virtual zones for each of the open and closed arms and the middle were created and total time spent with all four paws in each region, plus movement and velocity across the whole maze were analysed. Head‐dips, stretch attend postures and grooming were manually scored. A head dip is defined when a rat is on an open arm and peers over the platform edge such that it is head is fully off the platform and a stretch attend posture (SAP) is scored when the body of the rat is close to the floor and it is rear legs are in the closed arm whilst it investigates the open arms or middle section. Anxiety on the EPM is associated with more stretch attend postures, fewer head dips and increased defecation in addition to reduced open arm exploration.^25^ Faecal boli were also counted for each animal after each run, and for female rodents vaginal cytology was performed after testing on the maze to determine oestrus stage.

### 2.6 Open field test

The open field apparatus was a 100 × 100 cm square wooden arena with 30 cm high walls, painted black. Light levels were 25 lux at the centre of the maze, and 11.8 lux in the corners of the maze. Rats were habituated to the test room for at least 1 h, and were then placed, individually, in the arena adjacent to the middle of the south wall, with their head facing the wall. Between tests the arena was cleaned with 70% ethanol. Animal movements were recorded by a camera mounted 200 cm, above the arena. Ethovision with virtual zones dividing the arena into a central region centre (25cm^2^ located 25 cm from each other wall) and outer region (75 cm^2^, within 25 cm of each wall was used to analyse the recordings. Locomotor activity and thigmotaxis were assessed during a 10‐min session, in terms of the amount of time rodents spent in the central and outer regions, the distance moved (cm) and velocity of movement(cm/s) in the entire apparatus. Faecal boli were also counted for every rodent and for female rodents vaginal cytology was performed after testing on the maze to determine oestrus stage.

### 2.7 Acoustic startle response (ASR) and pre‐pulse inhibition (PPI)

ASR and PPI were assessed using a pair of R‐Lab™ Startle Response System chambers (San Diego Instruments, San Diego, USA). Each sound proofed chamber was equipped with a Perspex enclosure (10 cm diameter), with doors at either end, into which a rat was placed. This tube was located on a Perspex plinth, directly of a Piezoelectric sensor which register flexion and converted this to an electrical signal (ASR) that was monitored by a computer equipped with SR‐LAB startle software (San Diego Instruments, San Diego, USA). In the roof of the chamber, above the centre of the enclosure was a loudspeaker through which background noise (65 db) and trial stimuli were presented.

Methods were conducted as in Geyer et al.^26^ A session (30 min, 91 trials) consisted of a 5‐min period of habituation (at background noise) followed by two blocks of trials. Blocks 1 and 2 consisted of 13 pulse‐alone startle trials (40 min duration, at either 120 or 105 db) and 15 prepulse trials, five trials each at either 4, 8 or 16 db above background noise levels, intermixed on a pseudorandom schedule. A prepulse trial consisted of a stimulus (40 ms duration) followed by a startle stimulus, as above, (100 ms onset to onset delay). The third block of 18 trials of pulse‐alone startle trials, 3 of each at 70, 80, 90, 100, 110 and 120 db, pseudorandomly presented. The ASR to the first three pulse‐alone trials at 120 dB and 105 dB in blocks 1 and 2 were averaged and analysed as an index of emotional reactivity, mean ASR was calculated as the average of the remaining 10 pulse‐alone trials/block and response to prepulse trials at each intensity/block were also averaged. PPI was calculated as the proportional difference between mean ASR for pulse‐alone trials and mean prepulse response at each prepulse intensity. ASR for trials in Block 2 were meaned together at each intensity used. Background intensities 105 dB and 120 dB were used to mitigate against potential floor and ceiling effects. However, with no evidence of such effects, and the pattern or results observed at both intensities, only the findings for a background intensity of 120 dB are reported here. All ASR values were weight adjusted before analysis. As previously, rats were habituated to a room for at least 60 min before testing, and then transferred to the test room and individually placed into each enclosure within a test chamber. Enclosures were cleaned 70% ethanol between subjects.

### 2.8 Lick microstructure assessment

Rats were trained and tested in 16 custom made drinking chambers (Med Associated Inc., St Albans, USA). These were 30 × 13 × 13 cm (L × W × H), with steel grid flooring and white plastic walls. Sucrose was accessible through drinking spouts attached to 50 mL cylinders, which could be lowered through left or right apertures in the front wall of the chamber by hand. A contact sensitive lickometer registered the licks made by rats to the nearest 0.01 s once the bottle was available, and MED‐PC Software (Med Associates, Inc) recorded the data. Rats were trained across five consecutive days for 10 min each day to drink 8% sucrose solution from the spouts. During the first session the spout was left to protrude into the cage to encourage drinking, but after this the spout stopped just beyond the opening in the cage to minimise accidental contact. Once all rats were consistently drinking, the test phase began. During test rats drunk 4% and 16% sucrose solutions for 4 days each in an order counterbalanced for genotype. Rats were allocated to cages in alternating wild‐type/ *Dlg2* ^*+/−*^ order and the same drinking cage was used for each animal across the experiment. Half of the rats received 4 days of 16% followed by 4 days of 4% and the other half the reverse to implement the counterbalance. The amount of fluid consumed by each rat was measured by weighing the drinking bottle before and after each session. Solutions were made up daily on a weight/weight basis.

Mean consumption of sucrose (g) and mean lick cluster size for each rat were extracted from the record of licks for analysis. A cluster was defined as a set of licks, each separated by an interlick interval of no more than 0.5 s. This criterion was used by Davis and his co‐workers who pioneered this technique^27^, ^28^ and in many previous studies employing this assay to assess analogues of anhedonia.^29^, ^30^, ^31^, ^32^

### 2.9 Social preference test

This test utilised the same arena as the open field with two wire mesh chambers (22 cm diameter) weighed down with 2 kg weights placed diagonally in opposite corners of the arena. The distance between side walls and the chambers was 18 cm, and the distance between them diagonally always 35 cm. Light was 24.8 lux. Rats were placed in the experimental room where the arena was separated from them by a curtain for 1 h prior to testing commencing. Each individual rat was given 10 min to explore the arena with empty chambers then removed to a separate holding cage for 5 min, before being placed back in the arena for the 10‐min social preference test. In the social preference test the rat was presented with test one chamber contained an unknown conspecific (stranger rat) and the other chamber and unknown object. For each test animal a same‐sex wild‐type rat that had no prior contact with the test rat was used as the stranger. All stranger rats were habituated to the chambers for 10 min prior to testing. For analysis raw exploration times of the chambers (rodents directing their nose at the chamber at a distance of <2 cm) were used in addition to d2 discrimination ratio (Equation (1)). Discrimination ratio gives a readout of the difference in exploration time between the two stimuli without the confound of overall tendency to explore for long or short durations.

(1) $$D2=\frac{\text{stranger exploration time}-\text{object exploration time}}{\text{total exploration time}}$$

### 2.10 Phencyclidine (PCP) induced locomotion

To examine PCP‐induced changes to locomotor activity rats were placed in 58 × 45 × 60 cm (l × w × h) Perspex boxes and recorded with a camera placed 200 cm above the boxes. Four boxes were used simultaneously but 60 cm high barrier walls prevented rats from interacting with each other. Rats were placed in the boxes for a 30‐min habituation period before being injected subcutaneously with 5 mg/kg dose of PCP hydrochloride (in 0.9% [w/w] saline, Sigma‐Aldrich, UK) and returned to the same box for a further 90 min. Ethovision software was used to analyse movement and velocity for each rat throughout the habituation and post‐drug phases in 10 min blocks.

### 2.11 Statistical analysis

All statistical analysis was performed using JASP Version 0.14.1 (JASP Team 2020). For traditional null‐hypothesis significance testing *p* < 0.05 was considered statistically significant. For ANOVA analysis where Maunchly's test indicated sphericity was violated Greenhouse–Geisser corrected values are reported. Because interactions that included sex were non‐significant in all analyses that included sex as a factor, the effects of sex as a variable are not reported here but these can be seen in Supplement 1.

Traditional null‐hypothesis significance testing only assesses how unlikely the observed data is given the assumption of the null hypothesis, and thus *p* > 0.05 does not distinguish evidence for the null hypothesis from data insensitivity.^33^ In contrast, Bayesian tests calculate the relative probabilities of the null and alternative hypotheses, and thus allow assessment of whether the evidence is in favour of either hypothesis. In this body of work Bayesian statistics have been applied where traditional null‐hypothesis significance testing shows a non‐significant result for a key effect or interaction where a null result is potentially theoretically informative (in particular, evidence for a lack of a difference across genotypes).

Bayes factors relate to the ratio of probability for the observed data under a model based on the null hypothesis compared with a model based on some specified alternative. When represented as BF01 Bayes factors vary between 0 and infinity, where 1 indicates that the data do not favour either model more than the other, values greater than 1 indicate increasing evidence for the null over the alternative hypothesis and values less than 1 increasing evidence for the alternative over the null hypothesis. When using Bayes factors to decide whether there is substantial evidence for the null over the alternative, the following conventions suggested by Jeffreys et al.^34^ can be followed: a Bayes factor between 1 and 3 gives weak or anecdotal support to the null, a factor between 3 and 10 represents some supporting evidence, while a factor more than 10 indicates strong evidence for the null.

Bayes factors were calculated for factorial ANOVAs in the way described by Rouder, Morey, Speckman, and Province^35^ and Rouder, Morey, Verhagen, Swagman, and Wagenmakers^36^ and were implemented using JASP 0.14.1 and the default prior scale for fixed and random effects and reported as the analysis of effects—this gives a BF_exclusion_ which is equivalent to BF01 when averaging across models including the factor or interaction of interest. Bayes factors for t‐tests were calculated as described by Rouderet al.^37^ and implemented using JASP 0.14.1 with the default settings for the Cauchy prior distribution on effect size under the alternative hypothesis.

## 3 RESULTS

### 3.1 Protein and mRNA expression

**FIGURE 1:**
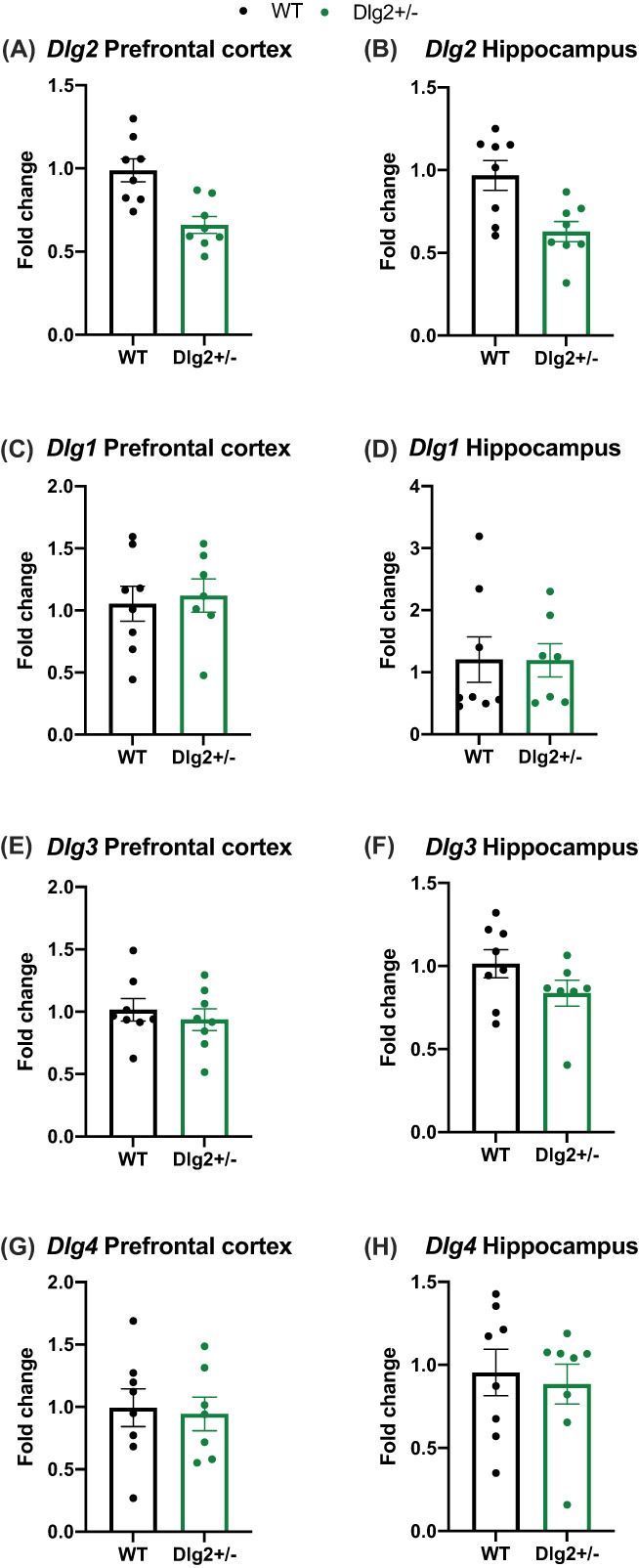
mRNA expression of *Dlg1‐4* in *Dlg2* ^*+/−*^ and wild‐type rats. Data is shown as mean ± SEM fold change plotted plus individual data points for *Dlg2* PFC (A), *Dlg2* hippocampus (B), *Dlg1* PFC (C), *Dlg1* hippocampus (D), *Dlg3* PFC (E), *Dlg3* hippocampus (F), *Dlg4* PFC (G) and *Dlg4* hippocampus (H). *n* = 8 wild‐type, 8 *Dlg2* ^*+/−*^. The *Dlg2* ^*+/−*^ rat shows a reduction of *Dlg2* expression in the hippocampus and prefrontal cortex compared to wild‐types, with no changes in the expression of *Dlg1*, *Dlg3* or *Dlg4*

mRNA expression for the *Dlgs* is shown in Figure 1 with summary values shown for the prefrontal cortex and hippocampus for *Dlg2* (A‐B), *Dlg1* (C‐D), *Dlg3* (E‐F) and *Dlg4* (G‐H). ΔCt was analysed by repeated measures ANOVA and Bayesian repeated measures ANOVA with repeated measures factors of brain region (prefrontal cortex, hippocampus) and between‐subjects factors of genotype. As Figure 1A and B show *Dlg2* expression varied with genotype (genotype main effect: *F* (1, 13) = 29.367, *p* < 0.001, *n* ^*2*^_*p*_ = 0.693) but did not vary with brain region (brain region main effect: *F* (1, 13) = 0.690, *p* = 0.421, *n* ^*2*^_*p*_ = 0.050; BF_exclusion_ = 2.049 and interaction: *F* (1, 13) = 0.031, *p* = 0.863, *n* ^*2*^_*p*_ = 0.002; BF_exclusion_ = 1.691).

As can be seen in Figure 1C and D the expression of *Dlg1* differed with brain region (*F* (1, 12) = 7.017, *p* = 0.021, *n* ^*2*^_*p*_ = 0.369) yet there were no genotype main effects (*F* (1, 12) = 0.498, *p* = 0.494, *n* ^*2*^_*p*_ = 0.040; BF_exclusion_ = 2.307) or interactions (*F* (1, 12) = 0.048, *p* = 0.831, *n* ^*2*^_*p*_ = 0.004; BF_exclusion_ = 1.650). This was much the same for *Dlg3* as shown in Figure 1E and F: brain region main effect (*F* (1, 12) = 11.150, *p* = 0.006, *n* ^*2*^_*p*_ = 0.482), with non‐significant results for genotype (*F* (1, 12) = 2.526, *p* = 0.138, *n* ^*2*^_*p*_ = 0.174; BF_exclusion_ = 1.307) and brain region × genotype (*F* (1, 12) = 0.528, *p* = 0.481, *n* ^*2*^_*p*_ = 0.042; BF_exclusion_ = 1.003). Figure 1G and H shows that for *Dlg4* there were no main effects of brain region (*F* (1, 12) = 1.397, *p* = 0.260, *n* ^*2*^_*p*_ = 0.104; BF_exclusion_ = 1.726), genotype (*F* (1, 12) = 0.155, *p* = 0.701, *n* ^*2*^_*p*_ = 0.013; BF_exclusion_ = 2.786) and no brain region × genotype interaction (*F* (1, 12) = 0.212, *p* = 0.653, *n* ^*2*^_*p*_ = 0.017; BF_exclusion_ = 3.852). This indicates that at the mRNA level there is no evidence of compensation for *Dlg2* decreases by changes in expression of other *Dlgs*.

**FIGURE 2:**
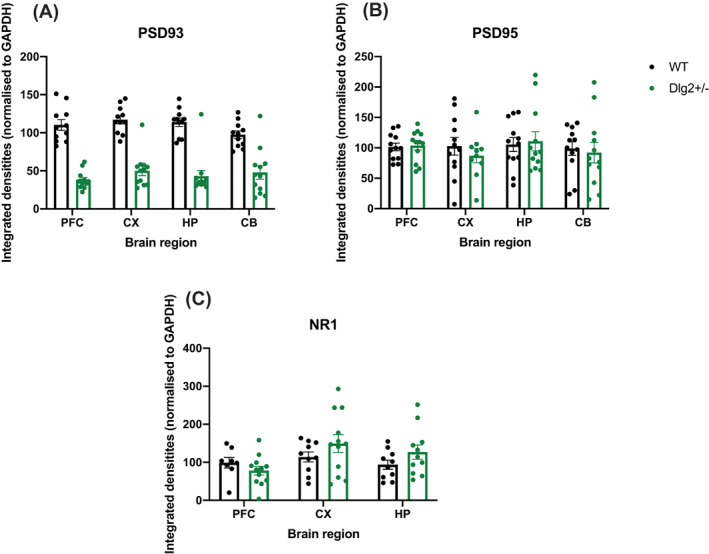
Expression of proteins PSD‐93 (A), PSD‐95 (B) and NR1 NMDA receptor subunit (C) in *Dlg2* ^*+/−*^ and wild‐type rats. These were assessed across four brain regions: prefrontal cortex (PFC), posterior cortex (CX), hippocampus (HP) and cerebellum (CB). Cerebellar NR1 expression was too low for analysis thus is not reported. Data is shown as mean ± SEM integrated density plotted plus individual data points. *n* = 12 wild‐type, 12 *Dlg2* ^*+/−*^. Across the prefrontal cortex, posterior cortex, hippocampus and cerebellum *Dlg2* ^*+/−*^ rats showed a decrease in PSD‐93 compared to wild‐types, with no changes in PSD‐95 or NR1 NMDA receptor subunit levels

Of the three proteins analysed only PSD‐93 showed consistent decreases across all four brain regions in the *Dlg2* ^*+/−*^ rats compared to wild‐types (Figure 2). Integrated densities were analysed using repeated measures ANOVA with within‐subjects factor of brain region (prefrontal cortex, posterior cortex, hippocampus, cerebellum—apart from NR1 where expression in cerebellum was negligible in all cases and thus this region was omitted from the analysis) and between‐subjects factor of genotype. Example blots can be seen in Supplementary Figure S1. The reduction of PSD‐93 in Dlg2+/− rats compared to wild‐types across all brain regions is shown in Figure 2A. Repeated measures ANOVA analysis for PSD‐93 showed a significant main effect of genotype (*F* (1, 22) = 13.680, *p* = 0.001, *n* ^2^_*p*_ = 0.383) demonstrating the success of the heterozygous gene knockout on reducing PSD93 protein levels. There was also a significant main effect of brain region (*F*(1.018, 22.400) = 20.893, *p* < 0.001, *n* ^2^_*p*_ = 0.487) and genotype × brain region interaction (*F*(1.018, 22.400) = 5.166, *p* = 0.032, *n* ^2^_*p*_ = 0.190). The significant genotype × brain region interaction was followed up with independent samples *t*‐tests. PSD‐93 was more abundant in the PFC of wild‐types than *Dlg2* ^*+/−*^ rats (*t* (22) = 6.057, *p* < 0.001, *d* = 2.473), likewise in the hippocampus (*t* (22) = 5.378, *p* < 0.001, *d* = 2.195), posterior cortex (*t* (22) = 4.032, *p* < 0.001, *d* = 1.646) and cerebellum (*t* (22) = 2.702, *p* = 0.013, *d* = 1.103).

PSD‐95 expression across genotypes and brain regions is shown in Figure 2B. Repeated measures ANOVA analysis of PSD‐95 levels showed no main effect of genotype with Bayes factor inconclusive (*F* (1, 22) = 3.805, *p* = 0.064, *n* ^2^_*p*_ = 0.147; BF_exclusion_ = 2.187), no main effect of brain region (*F*(1.722, 37.884) = 0.175, *p* = 0.808, *n* ^2^_*p*_ = 0.008; BF_exclusion_ = 18.288) and no genotype × brain region interaction with Bayes factors providing evidence for the null (*F*(1.722, 37.884) = 1.140, *p* = 0.324, *n* ^2^_*p*_ = 0.049; BF_exclusion_ = 23.396).

Similarly for analysis of NR1 NMDA receptor subunit levels (Figure 2C) there was no main effect of genotype (*F* (1, 17) = 0.262, *p* = 0.616, *n* ^2^_*p*_ = 0.015; BF_exclusion_ = 3.752), brain region (*F*(1.148, 19.514) = 0.884, *p* = 0.373, *n* ^2^_*p*_ = 0.049; BF_exclusion_ = 4.026) or genotype × brain region interaction (*F*(1.148, 19.514) = 0.902, *p* = 0.368, *n* ^2^_*p*_ = 0.050; BF_exclusion_ = 8.238). Thus, it seems that *Dlg2* haploinsufficiency does not have downstream effects on the expression of related proteins.

### 3.2 Behaviour in anxiety tests

**FIGURE 3:**
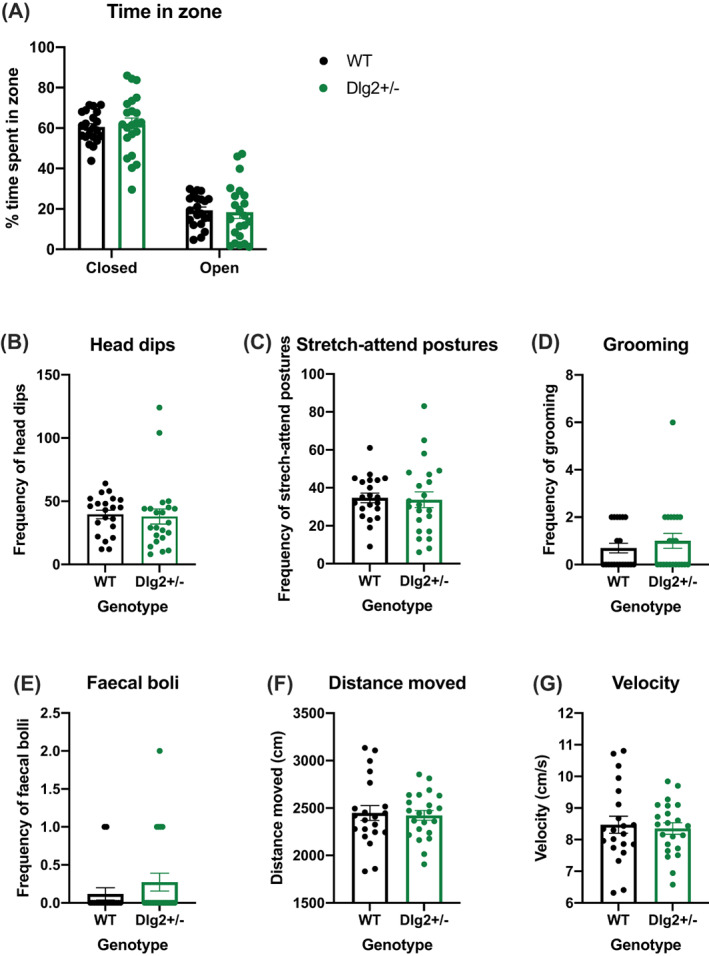
Effect of *Dlg2* heterozygous knockout on anxiety‐related behaviour in the elevated plus maze. Data is shown as mean ± SEM with data points representing individuals (A) time in zone (B) head dips (C) stretch‐attend postures (D) grooming (E) defecation (F) distance moved (G) velocity. *n* = 24 wild‐type, 21 *Dlg2* ^*+/−*^. *Dlg2* ^*+/−*^ rats performed comparably to wild‐types on elevated plus maze measures of anxiety and hyperactivity

Figure 3 shows measures from the EPM for time in closed and open arms (3A), head‐dips (3B), stretch‐attend postures (3C), grooming (3D), distance travelled (3E), velocity (3F) and defecation (3G). Where rodents spent their time in the maze was analysed using repeated measures ANOVA and Bayesian repeated measures ANOVA with within‐subjects factor of arm (closed, open) and between‐subjects factor of sex and genotype. Ethological measures and movement were analysed with ANOVA and Bayesian ANOVA with factors of sex and genotype. There were no sex effects on any EPM measures as reported in Supplementary Results S.2.1.1.

Figure 3A shows a general tendency across groups to avoid the open arms of the maze, however there were no differences between wild‐type and *Dlg2* ^*+/−*^ rats in the proportion of time spent in open and closed arms: main effect of zone (*F*(1.347, 52.518) = 158.103, *p* < 0.001, *n* ^2^_*p*_ = 0.802) in the absence of a main effect of genotype (*F* (1, 39) = 1.275, *p* = 0266, *n* ^2^_*p*_ = 0.032; BF_exclusion_ = 11.197) or zone × genotype interaction (*F*(1.347, 52.518) = 0.052, *p* = 0.887, *n* ^2^_*p*_ = 0.001; BF_exclusion_ = 17.680). There were also no genotype‐related difference in the ethological measures assessed: head‐dips (Figure 3B, F (1, 33) = 0.034, *p* = 0.854, *n* ^2^_*p*_ = 0.001; BF_exclusion_ = 4.266), stretch‐attend postures (Figure 3C, F (1, 33) = 0.190, *p* = 0.666, *n* ^2^_*p*_ = 0.006; BF_exclusion_ = 3.528), grooming (Figure 3D, F (1, 33) = 0.002, *p* = 0.961, *n* ^2^_*p*_ = 0.000; BF_exclusion_ = 3.816) or defecation (Figure 3E, F (1, 39) = 1.505, *p* = 0.227, *n* ^2^_*p*_ = 0.039; BF_exclusion_ = 2.471). There were also no genotype‐related effects on distance travelled as shown in Figure 3F (*F* (1, 33) = 0.082, *p* = 0.776, *n* ^2^_*p*_ = 0.002; BF_exclusion_ = 4.182) or velocity (Figure 3G, (*F* (1, 33) = 0.082, *p* = 0.776, *n* ^2^_*p*_ = 0.002; BF_exclusion_ = 4.151). These findings indicate that *Dlg2* ^*+/−*^ rats do not appear to have an anxiety phenotype in the EPM, although both wild‐type and *Dlg2* ^*+/−*^ rats demonstrated the expected anxiogenic profile for this test.

**FIGURE 4:**
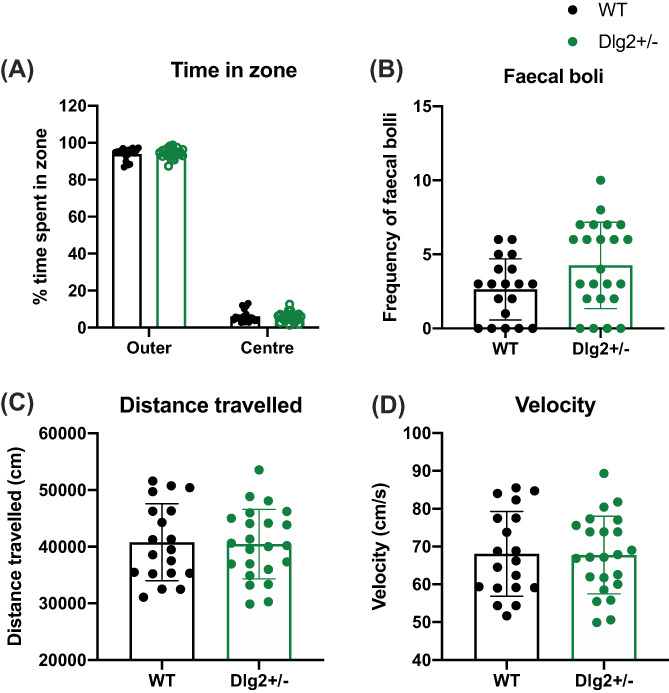
Effect of *Dlg2* heterozygous knockout on open‐field measures Data is shown as mean ± SEM plus individual values for (A) time in zone (B) velocity (C) distance travelled and (D) defection. *n* = 24 wild‐type, 21 *Dlg2* ^*+/−*^. *Dlg2* ^*+/−*^ rats performed comparably to wild‐types on open‐field measures of anxiety and hyperactivity

The distribution of time spent in maze zones was analysed using repeated measures ANOVA and Bayesian repeated measures ANOVA with within‐subjects factor of zone (central, outer) and between subjects factor of sex and genotype. Movement and defection were analysed with ANOVA and Bayesian ANOVA with factors of sex and genotype. As with EPM there were no sex effects and these are reported in Supplementary Results S.2.1.2. Figure 4 shows the results from the open field for time in the central and outer areas (4A), velocity (4B), distance moved (4C) and defecation (4D). Figure 4A demonstrates that while there was a general tendency to avoid the central region, there were no differences between wild‐type and *Dlg2* ^*+/−*^ rats in the proportion of time spent in centre and outer zones: main effect of zone (*F* (1, 38) = 9100.383, *p* < 0.001, *n* ^2^_*p*_ = 0.996) but no main effect of genotype (*F* (1, 38) = 0.697, *p* = 0.409, *n* ^2^_*p*_ = 0.018; BF_exclusion_ = 7.292) or zone × genotype interaction (*F* (1, 38) = 0.232, *p* = 0.633, *n* ^2^_*p*_ = 0.000; BF_exclusion_ = 6.064). While there was a suggestion that *Dlg2* ^*+/−*^ rats defecated more than wild‐type controls (Figure 4D), there was no significant effect of genotype (*F* (1, 38) = 3.769, *p* = 0.060, *n* ^2^_*p*_ = 0.090; BF_exclusion_ = 0.880). Although the BF was inconclusive here, it should be remembered that there was also no suggestion of a genotype‐related effect on defecation in the EPM. There were no genotype differences in either distance travelled (Figure 4C, F (1, 38) = 0.002, *p* = 0.961, *n* ^2^_*p*_ < 0.001; BF_exclusion_ = 3.551) or velocity (Figure 4B, F (1, 38) = 0.013, *p* = 0.908, *n* ^2^_*p*_ < 0.001; BF_exclusion_ = 3.753). Overall while the experiment demonstrated anxiety generally, with all the rats avoiding the aversive open central region, there were no genotype effects on this nor any other measure in the open field.

### 3.3 Acoustic startle response (ASR) and pre‐pulse inhibition (PPI)

**FIGURE 5:**
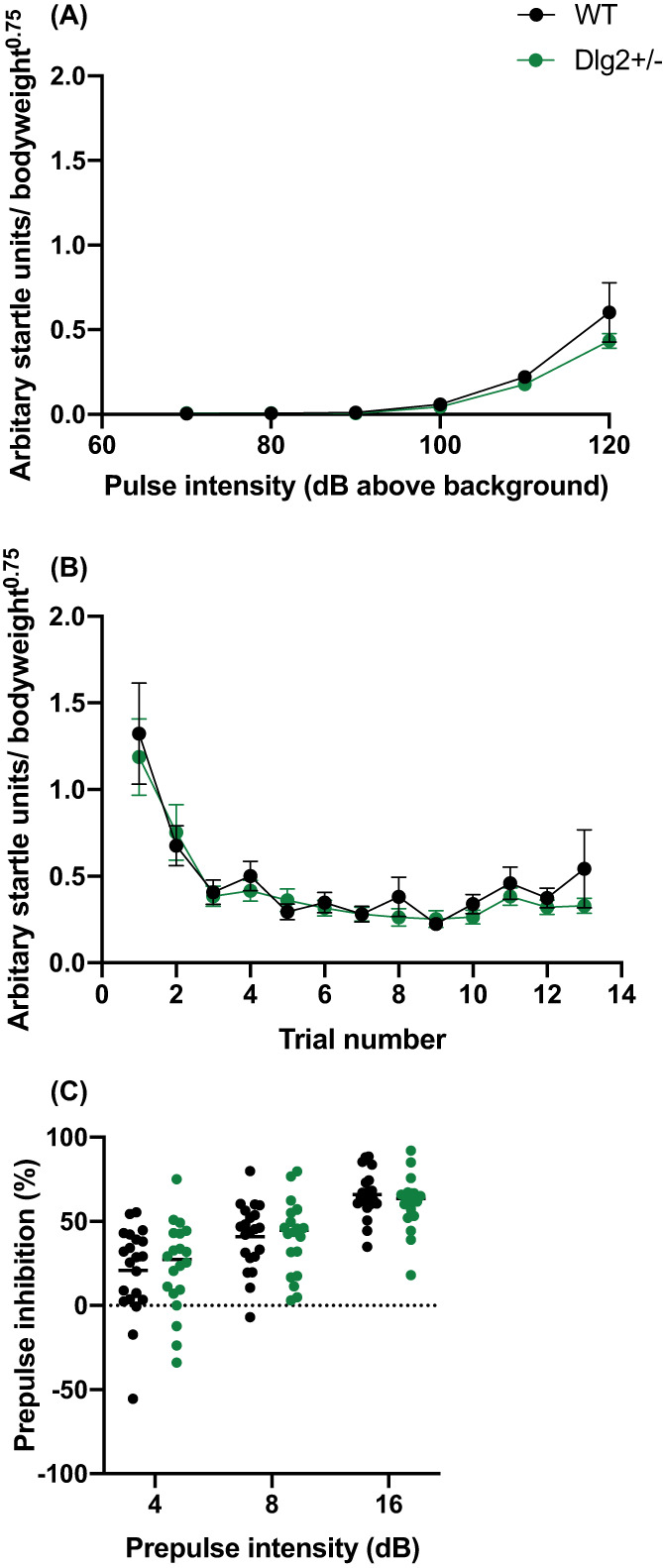
Effect of *Dlg2* heterozygosity on acoustic startle response and pre‐pulse inhibition. (A) Mean ± SEM weight‐adjusted ASR to 70–120 dB pulses above background. (B) Mean ± SEM habituation of startle response through increasing pulse trials and (C) Mean ± SEM PPI plus individual values with 4, 8 and 16 dB (above background) pre‐pulse. *n* = 24 wild‐type, 21 *Dlg2* ^*+/−*^. *Dlg2* ^*+/−*^ rats did not differ from wild‐types in their startle response, habituation of startle responding over trials or pre‐pulse inhibition

Response to increasing amplitudes of startle stimuli were assessed using mixed ANOVA and mixed Bayesian ANOVA with within‐subjects factor of pulse intensity (70, 80, 90, 100, 110 and 120 dB) and between‐subjects factors of genotype and sex from data acquired in the third block of trials from the startle sessions (Figure 5A). There was an absence of sex‐related effects on all ASR and PPI measures which are reported in Supplementary Information. There was a significant main effect of pulse intensity (*F*(1.089, 43.542) = 29.705, *p* < 0.001, *n* ^2^_*p*_ = 0.426) indicating an increased responding at higher startle intensities. There was no significant main effect of genotype (*F* (1, 40) = 0.872, *p* = 0.356, *n* ^*2*^_*p*_ = 0.021; BF_exclusion_ = 8.964), nor a genotype × pulse interaction (*F*(1.089, 43.542) = 0.590, *p* = 0.460, *n* ^2^_*p*_ = 0.015; BF_exclusion_ = 22.407). Thus, wild‐type and *Dlg2* ^*+/−*^ rats showed equal responding to increasing startle amplitudes, suggesting equivalent acoustic startle responses.

Habituation of the startle response (Figure 5B) to 120db pulse‐alone stimuli was assessed using mixed ANOVA and Bayesian mixed ANOVA with within‐subjects factors of trial number and between subjects factors of genotype and sex. As expected, the startle response habituated as trials progressed (main effect of trial: *F*(2.130, 85.190) = 13.127, *p* < 0.001, *n* ^2^_*p*_ = 0.247) but there were no differences between wild‐type and *Dlg2* ^*+/−*^ rats (genotype × trial interactions: *F*(2.130, 85.190) = 0.498, *p* = 0.621, *n* ^2^_*p*_ = 0.012; BF_exclusion_ = 643.328 or main effect of genotype: *F* (1, 40) = 0.347, *p* = 0.559, *n* ^2^_*p*_ = 0.009; BF_exclusion_ = 10.545).

There were also no differences between wild‐type and *Dlg2* ^*+/−*^ rats on pre‐pulse inhibition (Figure 5C). Repeated measures ANOVA and Bayesian repeated measures ANOVA with factors of pre‐pulse intensity (4, 8 and 16 dB above background), genotype and sex were used. There was generally greater inhibition of the startle response with increasing pre‐pulse intensity (significant main effect of pre‐pulse: *F*(2, 80) = 83.401, *p* < 0.001, *n* ^2^_*p*_ = 0.676), but there were no significant genotype × pre‐pulse interaction (*F*(2, 80) = 1.097, *p* = 0.339, *n* ^2^_*p*_ = 0.027; BF_exclusion_ = 5.712 or main effect of genotype (*F*(2, 80) = 0.011, *p* = 0.917, *n* ^2^_*p*_ = 0.000; BF_exclusion_ = 5.482) on PPI.

### 3.4 Lick microstructure assessment

**FIGURE 6:**
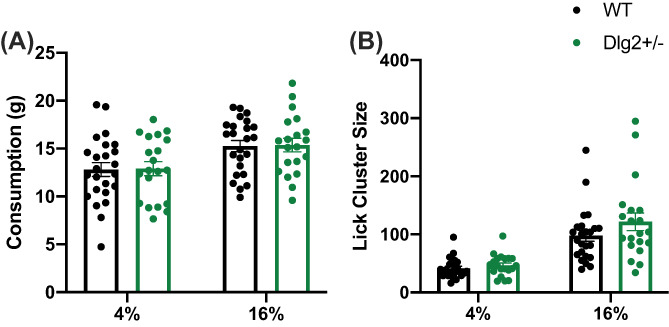
The drinking behaviour of *Dlg2* ^*+/−*^ and wild‐type rats when presented with low and high concentrations of sucrose. Data is shown as mean ± SEM plus individual values (A) consumption (g) and (B) lick cluster size. *n* = 28 wild‐type, 20 *Dlg2* ^*+/−*^. Consumption and lick cluster size to low and high sucrose solutions did not vary with genotype

Repeated measures ANOVA and Bayesian repeated measures ANOVA were used to analyse consumption and lick cluster data with genotype as a between subject factor and sucrose concentration as a within‐subject factor. As Figure 6A shows consumption of sucrose varied with concentration with rats consuming greater volumes of the 16% solution relative to 4% (main effect of concentration, *F* (1, 46) = 14.582, *p* = 0.015, *n* ^2^_*p*_ = 0.121). Genotype had no effect on sucrose consumption as shown by the non‐significant main effect of genotype, *F* (1, 46) = 0.055, *p* = 0.952, *n* ^2^_*p*_ = 0.000; BF_exclusion_ = 1.762. The genotype × concentration interaction was significant *F* (1, 46) = 7.429, *p* = 0.009, *n* ^2^_*p*_ = 0.139, reflecting the larger 4–16% based consumption change in the *Dlg2* ^*+/−*^ rats compared to the wild‐types.

Figure 6B reveals that lick cluster size also varied with sucrose concentration, as both wild‐type and *Dlg2* ^*+/−*^ rats showed larger cluster sizes at 16% than to less palatable 4% (main effect of concentration, *F* (1, 46) = 56.074, *p* < 0.001, *n* ^2^_*p*_ = 0.549). Genotype had no effect on lick cluster size at either concentration (non‐significant main effect of genotype, *F* (1, 46) = 0.642, *p* = 0.427, *n* ^*2*^_*p*_ = 0.014 non‐significant genotype × concentration interaction *F* (1, 46) = 0.002, *p* = 0.965, *n* ^2^_*p*_ = 0.000). Evidence for any genotype effect on lick cluster size is inconclusive (BF_exclusion_ = 2.384) as is that for the genotype × concentration interaction (BF_exclusion_ = 2.341). Because evidence for impaired hedonic reactions would require that *Dlg2* ^*+/−*^s would have lower lick cluster size than wild‐types Bayesian one‐tailed independent samples t‐tests were done on the lick cluster sizes for 4% and 16% conditions, finding evidence for the absence of this expected *Dlg2* ^*+/−*^ less than wild‐type effect in both instances (4% BF_01_ = 7.887, 16% BF_01_ = 5.735).

The variation in both lick cluster and consumption with concentration is expected and informs that the experiment successfully manipulated the hedonic properties of the stimuli. The lack of any reduction in lick cluster size for the *Dlg2* ^*+/−*^ rats suggests there is no suggestion of an anhedonic response to palatable stimuli.

### 3.5 Social preference test

**FIGURE 7:**
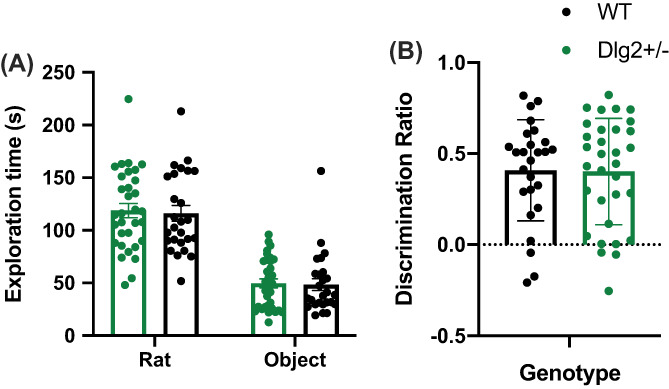
*Dlg2* ^*+/−*^ and wild‐type exploration times on the social preference task. Data is shown as mean ± SEM plus individual values (A) raw exploration and (B) d2 discrimination ratios. *n* = 26 wild‐type, 32 *Dlg2* ^*+/−*^. There was no effect of genotype on rodents preference for exploring the unknown conspecific relative to the object in the social preference task

Raw exploration times for conspecific and object in the social preference test are shown in Figure 7A. These data were analysed by mixed model ANOVA and Bayesian ANOVA with the within‐subject factor of item (conspecific, object) and between‐subjects factors of sex and genotype. The conspecific was explored more than the object (significant main effect of item:(*F* (1, 50) = 76.012, *p <* 0.001, *n* ^2^ *p* = 0.603) however this did not differ with genotype (non‐significant item × genotype interaction, *F* (1, 50) = 0.479, *p =* 0.492, *n* ^2^ *p* = 0.009; BF_exclusion_ = 7.678 or genotype main effect *F* (1, 50) = 0.014, *p =* 0.907, *n* ^2^ *p* = 0.000279; BF_exclusion_ = 7.986).

The discrimination ratio for social preference is shown in in Figure 7B. This was significantly different from 0 for the entire cohort (one‐sample t‐test *t (*53*) =* 9.988*, p <* 0.001*, d =* 1.359) reflecting the tendency to explore the conspecific more than the object. There were no genotype differences in discrimination ratio: non‐significant main effect of genotype (*F* (1, 50) = 0.850, *p =* 0.361, *n* ^2^ *p* = 0.017; BF_exclusion_ = 4.973). This reflects the fact that rats explored the conspecific more than the object as expected, yet *Dlg2* haploinsufficiency has no influence on this tendency.

### 3.6 PCP‐induced locomotion

**FIGURE 8:**
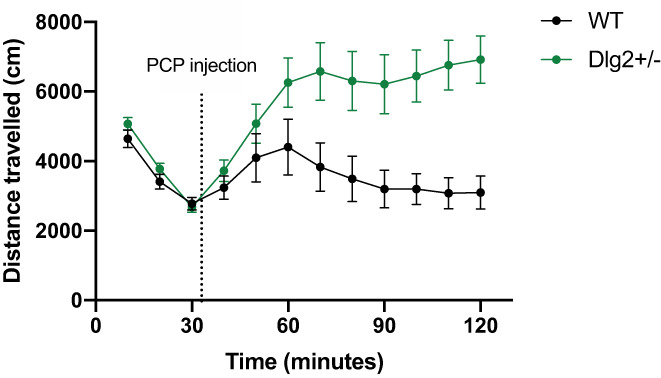
Locomotor activity in response to PCP injection in *Dlg2* ^*+/−*^ and wild‐type rats. Data is shown as mean ± SEM distance travelled plotted in 10‐min bins. The dotted line just past 30 min denotes when the PCP injection occurred. *n* = 26 wild‐type, 32 *Dlg2* ^*+/−*^. *Dlg2* ^*+/−*^ rats demonstrated a locomotor response to PCP which was more sustained and exaggerated than wild‐types

Figure 8 shows the distance travelled in the arena over the 120‐min test period (30 mins preceding 5 mg/kg PCP administration and the following 90 mins) for wild‐type and *Dlg2* ^*+/−*^ rats. Distance travelled was analysed separately for the 30 min preceding injection and the 90 min post injection using repeated measures ANOVA with the repeated measures factor of time bin (3 × 10‐min bins covering the 30 min preceding injection and 9 × 10‐min bins 90 min post‐injection) and between‐subjects factors of sex and genotype.

When analysing distance travelled for the 30 min before the injection of 5 mg/kg PCP, there was a main effect of time bin (*F*(2, 98) = 227.098, *p <* 0.001, *n* ^2^_*p*_ = 0.823) as activity decreased through rats initial habituation. There was no main effect of genotype (*F* (1, 49) = 0.452, *p* = 0.505, *n* ^2^_*p*_ = 0.009; BF_exclusion_ = 2.165) but the time bin × genotype interaction approached significance (*F*(2, 98) = 2.516, *p* = 0.086, *n* ^2^_*p*_ = 0.049; BF_exclusion_ = 1.243) with *Dlg2* ^*+/−*^ rats showing a slight tendency to a faster reduction in activity across the 30 min pre‐injection habituation period. At 30 min there was no difference in activity between genotypes (*t* (51) = −0.420, *p* = 0.676, *d* = −0.116; BF_01_ = 3.171), providing an equivalent baseline before PCP injection. Comparisons for genotype at 10 min (*t* (52) = 1.375, *p* = 0.175, *d* = 0.375; BF_01_ = 2.359) and 20 min (*t* (51) = 1.375, *p* = 0.175, *d* = 0.379; BF_01_ = 1.721) were also non‐significant.

Following injection of PCP, there was a significant increase in movement by both wild‐type and *Dlg2* ^*+/−*^ rats (main effect of time bin: *F*(2.829, 141.460) = 4.044, *p* = 0.010, *n* ^2^_*p*_ = 0.075), although the effect was much greater and longer lasting in *Dlg2* ^*+/−*^ rats, as demonstrated by a significant time bin × genotype interaction (*F*(2.829, 141.460) = 5.125, *p* = 0.003, *n* ^2^_*p*_ = 0.093) and main effect of genotype (*F* (1, 50) = 9.873, *p* = 0.003, *n* ^2^_*p*_ = 0.165). Follow up analyses of the time bin by genotype interaction indicated that the *Dlg2* ^*+/−*^ animals were more active than wild‐types on time bin 70 and onwards (smallest *t*(56) = 2.466, *p* = 0.017, *d* = 0.651) but not on time bin 60 and before (largest *t*(56) = 1.738, *p* = 0.088, *d* = 0459).

## 4 DISCUSSION

This work presents the characterisation of which molecular and behavioural capabilities are spared and impaired in the *Dlg2* heterozygous rat model; a model with direct clinical relevance to CNVs that increase risk for a variety of psychiatric conditions including schizophrenia,^3^ autism^1^ and intellectual disability.^2^ The model is specific to *Dlg2* and valid, with evidence that mRNA and protein expression of *Dlg2* is reduced in the absence of changes to levels of other *Dlgs*. Behaviourally *Dlg2* ^*+/−*^ rats performed comparably to wild‐types on tests of anxiety, hedonic reactions, social behaviour, and sensorimotor gating.

When locomotor response to PCP challenge was assessed, *Dlg2* ^*+/−*^ rats demonstrated a potentiated response to the drug. This demonstrates the first behavioural correlate of *Dlg2* heterozygosity in the rat model and is in line with electrophysiological data demonstrating a change in NMDAR function in *Dlg2* heterozygous rats.^27^ This contrasts with work on *Dlg2* homozygous knockdown models where no change in NMDAR function has been documented,^38^, ^39^, ^40^ however Zhang et al^17^ observed that PSD‐93 deficiency in cortical neurons reduces the expression of NR2A and NR2B NMDAR subunits and changes Ca^2+^ influx through NMDARs.

Altered psychostimulant sensitivity has also been shown in other psychosis‐relevant CNV rodent models, the 22q11.2^41^ and 1q21.1^42^ microdeletion mouse models. In the 22q11.2 mouse this manifested as an exaggerated locomotor response to PCP and ketamine. In the 1q21.1 mouse exaggerated locomotor behaviour was seen in response to amphetamine but was not significant with administration of PCP, yet PCP resulted in sensorimotor gating impairments. As administration of non‐competitive NMDAR antagonists in healthy rodents and humans can induce a behavioural syndrome isomorphic to positive and negative symptoms of schizophrenia^22^, ^43^, ^44^ and exacerbates positive and negative symptoms in schizophrenic patients^45^, ^46^, ^47^ the finding of PCP sensitivity in these rodent CNV models may highlight a general psychosis susceptibility. Findings such as these contribute to the hypoglutamaterigic hypothesis of psychosis.^48^ However, it is difficult to use these findings to determine which biological processes are altered in CNV rodent models. Acute PCP administration has been reported to activate serotonergic, glutamatergic, noraderenergic, cholinergic and neurotensinergic transmission in rodents and monkeys.^49^, ^50^, ^51^ It will be informative to focus on investigating how individual behavioural alterations to NMDAR antagonists, and their timescales, may be subserved by particular neurotransmitter dysfunctions to identify mechanisms underpinning these in genetic disorder models.

Coherence between findings on homozygous mouse models, heterozygous mouse models and the CRISPR‐Cas9 generated *Dlg2* ^*+/−*^ rat were mixed. Findings of impaired social preference and hypoactivity in the open field test which are seen in *Dlg2* ^*−/−*^ mice but not *Dlg2* ^*+/−*^ mice^14^, ^15^ were also not seen in the *Dlg2* ^*+/−*^ rat. Comparisons between complete and heterozygous gene knockout models allow a distinction to be made between knowledge about the function of a protein and processes which require complete PSD93 levels. The difference here implies that having some functional PSD93 might ‘rescue’ these phenotypes. This could be supported by PSD93 acting in tandem with PSD95 or other MAGUKs. Where social behaviour is concerned it has been shown that there is a similarity in social deficits in *PSD95* ^*+/−*^ mice and *PSD93* ^*−/−*^ mice, with increased expression of PSD93 in the hippocampus of *PSD95* ^*+/−*^ mice implying that PSD93 is acting to compensate in this mouse model.^15^ In *Dlg2* ^*+/−*^ rodent models it could be that intact PSD95 in the presence of some PSD93 was sufficient to support intact social preference performance, meaning that while PSD93 has some role in social behaviours it is not so essential that genetic haploinsufficiency produces a gross deficit.

The increased self‐grooming phenotype seen in homozygous and heterozygous mouse *Dlg2* mutants^15^ was not seen in the rat model. This comes with the caveat that grooming was assessed in the EPM and open‐field test in the rat line, while Yoo et al^14^ assessed grooming in a clean home cage. This comprised 20 min of habituation followed by 10 test minutes in which self‐grooming was recorded. *Dlg2* ^*+/−*^ and wild‐type rats placed in the EPM or open field for 5 and 10 min, respectively, would not have habituated to the apparatus, as is crucial for anxiety tests, meaning that increased self‐grooming may only be seen after the habituation period.

The observed lack of anxiety and pre‐pulse inhibition phenotypes in the *Dlg2* ^*+/−*^ rat were also found in *Dlg2* ^*+/−*^ mice.^52^ However, *Dlg2* ^*+/−*^ mice demonstrated a subtle deficit of habituation to the acoustic stimulus in this facet of the sensorimotor gating task, which was not seen in the *Dlg2* ^*+/−*^ rat. This may be due to differences in model generation, with the heterozygous mouse generated by introduction of a cassette upstream of the critical exon (14) on chromosome 7 while the heterozygous rat was generated by 7 bp deletion within the rat *Dlg2* gene resulting in a frame shift and premature stop codon. This behavioural difference also points to potential caveats in comparisons across psychiatric risk models with different species backgrounds, which is also relevant when comparing homozygous and heterozygous mice with the rat model.

Another point of interest is the lack of interactions between sex and genotype in this work (see Supplement 1 for detailed data), which in the main was conducted on mixed sex cohorts. This is an important inclusion in the rodent modelling of psychiatric susceptibility literature which is often conducted on male‐only cohorts, and thus runs the risk of limited translational generalisability. Critically, the results here relating to the genotype manipulation were unaffected by the sex of the animals.

## 5 CONCLUSION

The *Dlg2* ^*+/−*^ rat validly models a single copy deletion of *Dlg2* including concomitant mRNA and protein decrease in the absence of obvious compensation. No gross behavioural deficits on tasks relevant to a broad spectrum of psychiatric phenotypes were found, except for exaggerated hyperlocomotion in response to PCP, an NMDAR‐antagonist. A similar selectivity in phenotype is seen for other psychiatric CNV models such as the 1q21.1 mouse.^42^ This demonstrates the behavioural subtlety of the model and highlights issues with drawing clinical conclusions from homozygous models. It also paves the way for investigation into more complex behavioural domains such as memory and learning without the concern of confounds from anxiety, hedonic processing, hearing, and social processing.

## CONFLICT OF INTEREST

The authors confirm there are no conflicts of interest to declare.

## Supporting information


**Appendix** S1: Supporting Information

## Data Availability

The data that support the findings of this study are available from the corresponding author upon reasonable request.

## References

[1] Ruzzo EK , Pérez‐Cano L , Jung JY , et al. Inherited and De Novo genetic risk for autism impacts shared networks. Cell. 2019;178(4):850‐866.e26. doi:10.1016/j.cell.2019.07.015 31398340 PMC7102900

[2] Reggiani C , Coppens S , Sekhara T , et al. Novel promoters and coding first exons in DLG2 linked to developmental disorders and intellectual disability. Genome med. 2017;9(1):1‐20. doi:10.1186/s13073-017-0452-y 28724449 PMC5518101

[3] Kirov G , Pocklington AJ , Holmans P , et al. De novo CNV analysis implicates specific abnormalities of postsynaptic signalling complexes in the pathogenesis of schizophrenia. Mol Psychiatry. 2012;17(2):142‐153. doi:10.1038/mp.2011.154 22083728 PMC3603134

[4] Egger G , Roetzer KM , Noor A , et al. Identification of risk genes for autism spectrum disorder through copy number variation analysis in Austrian families. Neurogenetics. 2014;15:117‐127. doi:10.1007/s10048-014-0394-0 24643514

[5] Noor A , Lionel AC , Cohen‐Woods S , et al. Copy number variant study of bipolar disorder in Canadian and UKpopulations implicates synaptic genes. Am J med Genet Part B Neuropsychiatr Genet. 2014;165(4):303‐313. doi:10.1002/ajmg.b.32232 24700553

[6] Gao K , Zhang Y , Zhang L , et al. Large De Novo Microdeletion in epilepsy with intellectual and developmental disabilities, with a systems biology analysis. Adv Neurobiol. 2018;21:247‐266. doi:10.1007/978-3-319-94593-4_9 30334225

[7] Irie M , Hata Y , Takeuchi M , et al. Binding of neuroligins to PSD‐95. Science. 1997;277(5331):1511‐1515. doi:10.1126/SCIENCE.277.5331.1511 9278515

[8] Niethammer M , Kim E , Sheng M . Interaction between the C terminus of NMDA receptor subunits and multiple members of the PSD‐95 family of membrane‐associated guanylate kinases. J Neurosci. 1996;16(7):2157‐2163. doi:10.1523/JNEUROSCI.16-07-02157.1996 8601796 PMC6578538

[9] Chen B , Gray J , Sanz‐Clemente A , et al. SAP102 mediates synaptic clearance of NMDA receptors. Cell Rep. 2012;2(5):1120‐1128. doi:10.1016/J.CELREP.2012.09.024 23103165 PMC3513525

[10] Frank RAW , Komiyama NH , Ryan TJ , Zhu F , O'Dell TJ , Grant SGN . NMDA receptors are selectively partitioned into complexes and supercomplexes during synapse maturation. Nat Commun. 2016;7(1):1‐13. doi:10.1038/ncomms11264 PMC5227094 27117477

[11] Dakoji S , Tomita S , Karimzadegan S , Nicoll R , Bredt D . Interaction of transmembrane AMPA receptor regulatory proteins with multiple membrane associated guanylate kinases. Neuropharmacology. 2003;45(6):849‐856. doi:10.1016/S0028-3908(03)00267-3 14529722

[12] Inanobe A , Fujita A , Ito M , Tomoike H , Inageda K , Kurachi Y . Inward rectifier K+ channel Kir2.3 is localized at the postsynaptic membrane of excitatory synapses. Am J Physiol Cell Physiol. 2002;282(6):C1396‐C1403. doi:10.1152/AJPCELL.00615.2001 11997254

[13] Leonoudakis D , Conti LR , Anderson S , et al. Protein trafficking and anchoring complexes revealed by proteomic analysis of inward rectifier potassium channel (Kir2.X)‐associated proteins *. J Biol Chem. 2004;279(21):22331‐22346. doi:10.1074/JBC.M400285200 15024025

[14] Yoo T , Kim SG , Yang SH , Kim H , Kim E , Kim SY . A DLG2 deficiency in mice leads to reduced sociability and increased repetitive behavior accompanied by aberrant synaptic transmission in the dorsal striatum. Mol Autism. 2020;11(19). doi:10.1186/s13229-020-00324-7 PMC7069029 32164788

[15] Winkler D , Daher F , Wüstefeld L , et al. Hypersocial behavior and biological redundancy in mice with reduced expression of PSD95 or PSD93. Behav Brain Res. 2018;352:35‐45. doi:10.1016/j.bbr.2017.02.011 28189758

[16] Nithianantharajah J , Komiyama NH , McKechanie A , et al. Synaptic scaffold evolution generated components of vertebrate cognitive complexity. Nat Neurosci. 2013;16(1):16‐24. doi:10.1038/nn.3276 23201973 PMC4131247

[17] Zhang M , Xu J‐T , Zhu X , et al. PSD‐93 deficiency protects cultured cortical neurons from NMDA receptor‐Triggered neurotoxicity. Neuroscience. 2010;166(4):1083‐1090. doi:10.1016/j.neuroscience.2010.01.030 20097270 PMC2846081

[18] Mena A , Ruiz‐Salas JC , Puentes A , Dorado I , Ruiz‐Veguilla M , De la Casa LG . Reduced prepulse inhibition as a biomarker of schizophrenia. Front Behav Neurosci. 2016;10(Oct):202. doi:10.3389/fnbeh.2016.00202 27803654 PMC5067522

[19] Wan L , Thomas Z , Pisipati S , Jarvis SP , Boutros NN . Inhibitory deficits in prepulse inhibition, sensory gating, and antisaccade eye movement in schizotypy. Int J Psychophysiol. 2017;114:47‐54. doi:10.1016/j.ijpsycho.2017.02.003 28189549

[20] Sinclair D , Oranje B , Razak KA , Siegel SJ , Schmid S . Sensory processing in autism spectrum disorders and fragile X syndrome—from the clinic to animal models. Neurosci Biobehav Rev. 2017;76(Pt B):235‐253. doi:10.1016/j.neubiorev.2016.05.029 27235081 PMC5465967

[21] Powell SB , Weber M , Geyer MA . Genetic models of sensorimotor gating: relevance to neuropsychiatric disorders. Curr Top Behav Neurosci. 2012;12:251. doi:10.1007/7854_2011_195 22367921 PMC3357439

[22] Javitt DC , Zukin SR . Recent advances in the phencyclidine model of schizophrenia. Am J Psychiatry. 1991;148(10):1301‐1308. doi:10.1176/ajp.148.10.1301 1654746

[23] Geyer MA , Ellenbroek B . Animal behavior models of the mechanisms underlying antipsychotic atypicality. Prog Neuro‐Psychopharmacol Biol Psychiatry. 2003;27(7):1071‐1079. doi:10.1016/j.pnpbp.2003.09.003 14642967

[24] Griesius S , O'Donnell C , Waldron S , et al. Reduced expression of the psychiatric risk gene DLG2 (PSD93) impairs hippocampal synaptic integration and plasticity. bioRxiv. 2021;08:454736. doi:10.1101/2021.08.02.454736 PMC9117295 35115661

[25] Humby T , Cross ES , Messer L , Guerrero S , Davies W . A pharmacological mouse model suggests a novel risk pathway for postpartum psychosis. Psychoneuroendocrinology. 2016;74:363‐370. doi:10.1016/j.psyneuen.2016.09.019 27728876 PMC5094271

[26] Geyer MA , Wilkinson LS , Humby T , Robbins TW . Isolation rearing of rats produces a deficit in prepulse inhibition of acoustic startle similar to that in schizophrenia. Biol Psychiatry. 1993;34(6):361‐372. doi:10.1016/0006-3223(93)90180-L 8218603

[27] Davis JD , Perez MC . Food deprivation‐ and palatability‐induced microstructural changes in ingestive behavior. Am J Physiol Regul Integr Comp Physiol. 1993;264(1):R97–R103. doi:10.1152/ajpregu.1993.264.1.r97 8430892

[28] Davis JD , Smith GP . Analysis of the microstructure of the rhythmic tongue movements of rats ingesting maltose and sucrose solutions. Behav Neurosci. 1992;106(1):217‐228. http://www.ncbi.nlm.nih.gov/pubmed/1554433 1554433

[29] Austen JM , Sprengel R , Sanderson DJ . GluA1 AMPAR subunit deletion reduces the hedonic response to sucrose but leaves satiety and conditioned responses intact. Sci Rep. 2017;7(1):1‐14. doi:10.1038/s41598-017-07542-9 28785046 PMC5547105

[30] Clarkson JM , Dwyer DM , Flecknell PA , Leach MC , Rowe C . Handling method alters the hedonic value of reward in laboratory mice. Sci Rep. 2018;8:1‐8. doi:10.1038/S41598-018-20716-3. 29402923 PMC5799408

[31] Wright RL , Gilmour G , Dwyer DM . Wistar Kyoto rats display Anhedonia in consumption but retain some sensitivity to the anticipation of palatable solutions. Front Behav Neurosci. 2020;14:70. doi:10.3389/FNBEH.2020.00070/FULL 32581735 PMC7283460

[32] Dwyer DM . Licking and Liking: The Assessment of Hedonic Responses in Rodents. Quar J Exp Psychol. 2012;65(3):371–394. 10.1080/17470218.2011.652969 22404646

[33] Dienes Z . Using Bayes to get the most out of non‐significant results. Front Psychol. 2014;5:781. doi:10.3389/fpsyg.2014.00781 25120503 PMC4114196

[34] Jeffreys H . Theory of Probability. 3rd ed. Oxford University Press; 1961.

[35] Rouder JN , Morey RD , Speckman PL , Province JM . Default Bayes factors for ANOVA designs. J Math Psychol. 2012;56(5):356‐374. doi:10.1016/J.JMP.2012.08.001

[36] Rouder JN , Morey RD , Verhagen J , Swagman AR , Wagenmakers EJ . Bayesian analysis of factorial designs. Psychol Methods. 2017;22(2):304‐321. doi:10.1037/MET0000057 27280448

[37] Rouder JN , Speckman PL , Sun D , Morey RD , Iverson G . Bayesian t tests for accepting and rejecting the null hypothesis. Psychon Bull Rev. 2009;16(2):225‐237. doi:10.3758/PBR.16.2.225 19293088

[38] Elias GM , Funke L , Stein V , Grant SG , Bredt DS , Nicoll RA . Synapse‐specific and developmentally regulated targeting of AMPA receptors by a family of MAGUK scaffolding proteins. Neuron. 2006;52(2):307‐320. doi:10.1016/j.neuron.2006.09.012 17046693

[39] Krüger JM , Favaro PD , Liu M , et al. Differential roles of postsynaptic density‐93 isoforms in regulating synaptic transmission. J Neurosci. 2013;33(39):15504‐15517. doi:10.1523/JNEUROSCI.0019-12.2013 24068818 PMC3782625

[40] Favaro PD , Huang X , Hosang L , et al. An opposing function of paralogs in balancing developmental synapse maturation. PLoS Biol. 2018;16(12):1‐41. doi:10.1371/journal.pbio.2006838 PMC6324823 30586380

[41] Didriksen M , Fejgin K , Nilsson SRO , et al. Persistent gating deficit and increased sensitivity to NMDA receptor antagonism after puberty in a new mouse model of the human 22q11.2 microdeletion syndrome: a study in male mice. J Psychiatry Neurosci. 2017;42(1):48–58. doi:10.1503/jpn.150381 27391101 PMC5373712

[42] Nielsen J , Fejgin K , Sotty F , et al. A mouse model of the schizophrenia‐associated 1q21.1 microdeletion syndrome exhibits altered mesolimbic dopamine transmission. Psychiatry. 2017;7:1261. doi:10.1038/s41398-017-0011-8 PMC5802512 29187755

[43] Snyder SH . Phencyclidine. Nature. 1980;285(5764):355‐356. doi:10.1038/285355a0 7189825

[44] Tamminga CA . Schizophrenia and glutamatergic transmission. Crit Rev Neurobiol. 1998;12(1–2):21‐36. doi:10.1615/CritRevNeurobiol.v12.i1-2.20 9444480

[45] Itil T , Keskiner A , Kiremitci N , Holden JM . Effect of phencyclidine in chronic schizophrenics. Can Psychiatr Assoc J. 1967;12(2):209‐212. doi:10.1177/070674376701200217 6040448

[46] Lahti AC , Koffel B , Laporte D , Tamminga CA . Subanesthetic doses of ketamine stimulate psychosis in schizophrenia. Neuropsychopharmacology. 1995;13(1):9‐19. doi:10.1016/0893-133X(94)00131-I 8526975

[47] Malhotra AK , Pinals DA , Adler CM , et al. Ketamine‐induced exacerbation of psychotic symptoms and cognitive impairment in neuroleptic‐free schizophrenics. Neuropsychopharmacology. 1997;17(3):141‐150. doi:10.1016/S0893-133X(97)00036-5 9272481

[48] Moghaddam B , Javitt D . From revolution to evolution: the glutamate hypothesis of schizophrenia and its implication for treatment. Neuropsychopharmacology. 2012;37(1):4‐15. doi:10.1038/npp.2011.181 21956446 PMC3238069

[49] Deutch AY , Tam SY , Freeman AS , Bowers MB , Roth RH . Mesolimbic and mesocortical dopamine activation induced by phencyclidine: contrasting pattern to striatal response. Eur J Pharmacol. 1987;134(3):257‐264. doi:10.1016/0014-2999(87)90356-6 3569414

[50] Hertel P , Mathé JM , Nomikos GG , Iurlo M , Mathé AA , Svensson TH . Effects of d‐amphetamine and phencyclidine on behavior and extracellular concentrations of neurotensin and dopamine in the ventral striatum and the medial prefrontal cortex of the rat. Behav Brain Res. 1995;72(1–2):103‐114. doi:10.1016/0166-4328(96)00138-6 8788863

[51] Jentsch JD , Elsworth JD , Redmond DE , Roth RH . Phencyclidine increases forebrain monoamine metabolism in rats and monkeys: modulation by the isomers of HA966. J Neurosci. 1997;17(5):1769‐1775. doi:10.1523/jneurosci.17-05-01769.1997 9030635 PMC6573388

[52] Pass R , Haan N , Humby T , Wilkinson LS , Hall J , Thomas KL . Selective behavioural impairments in mice heterozygous for the cross disorder psychiatric risk gene DLG2. bioRxiv. 2021;10:463181. doi:10.1101/2021.10.05.463181 PMC9393930 35118804

